# A34 GLUTEN-FREE DIET IMPROVES DYSPEPTIC SYMPTOMS IN PATIENTS WITH TYPE 1 DIABETES IN WHOM CELIAC DISEASE WAS EXCLUDED

**DOI:** 10.1093/jcag/gwab049.033

**Published:** 2022-02-21

**Authors:** P M Miranda, G H Rueda, C Seiler, M I Pinto-Sanchez, P Bercik

**Affiliations:** 1 Farncombe Institute, McMaster University, Hamilton, ON, Canada; 2 Medicine, McMaster University, Hamilton, ON, Canada; 3 Medical Sciences, McMaster University, Oakville, ON, Canada

## Abstract

**Background:**

Patients with type 1 Diabetes Mellitus (T1DM) often suffer from dyspeptic symptoms, such as abdominal pain, bloating, early satiety, nausea and vomiting. T1DM shares genetic risk factors (HLA-DQ2 and DQ8) with celiac disease, an autoimmune disorder caused by an immune reaction to gluten. Patients with concomitant T1DM and celiac disease benefit from a gluten-free diet (GFD), as it improves their symptoms, gastric emptying and small intestinal inflammation. However, it is unknown whether GFD has any benefit in patients with T1DM without celiac disease, who present with symptoms of dyspepsia or gastroparesis.

**Aims:**

To investigate the role of a GFD in the management of moderate to severe dyspeptic symptoms in non-celiac patients with T1DM.

**Methods:**

We enrolled 13 adult T1DM patients, in whom celiac disease was ruled out, suffering from two or more upper GI symptoms. The patients were instructed to adhere to a strict GFD for a period of 1 month, under the supervision of a dietitian. Glycemic levels were monitored by a continuous glucose monitoring device (CGM) for 2 weeks before, and at the end of the GFD period. Standardized questionnaires were used to assess upper GI symptoms (Gastroparesis Cardinal Symptoms Index, Short Form Leeds Dyspepsia questionnaire), general quality of life (Patient Assessment of Upper GI Disorders Quality of Life), and anxiety and depression (Hospital Anxiety Depression scale). Blood samples were collected to assess glycaemia (Hb1Ac) and immune markers. Scintigraphy and videofluoroscopy were used to assess gastric emptying.

**Results:**

After one month on a GFD, T1DM patients reported a significant improvement in nausea (p<0.05), sensation of fullness (p<0.001) and bloating (p<0.0001). Overall dyspepsia symptoms also improved (p<0.01), with 92% of patients reporting decreased dyspepsia scores. Moreover, there was an improvement in the quality of life (p<0.01), and decreased scores of anxiety and depression (p<0.01 and p<0.05, respectively). There were no changes in gastric emptying or glycemic management metrics, such as mean glucose level, time in target, glucose variance or HbA1c levels. Interestingly, one year after the end of the study, 63.6% of participants continued to follow a GFD. The most common reason to remain on a GFD reported by these patients was “because it improved GI symptoms”. Questionnaire data collected at the follow-up demonstrated that the improvement of dyspeptic symptoms persisted after 1 year on GFD (p<0.001).

**Conclusions:**

GFD improves dyspeptic symptoms in T1D patients without concomitant celiac disease, without affecting their glycemic levels or gastric emptying. Although these results need to be validated in a larger study, our data suggest that GFD could be used as a therapeutical tool for T1DM patients grappling with burdensome upper GI symptoms.

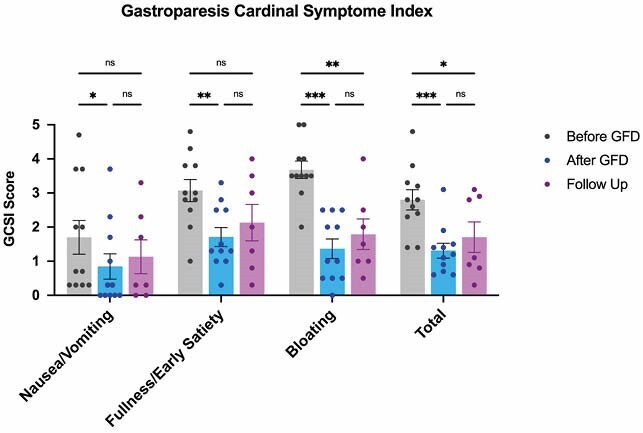

Gluten-Free diet improves dyspepsia symptoms in Type 1 Diabetes patients, after 1 month and 1 year

**Funding Agencies:**

CIHR

